# Vulnerability in Children with Celiac Disease: A Scoping Review Protocol

**DOI:** 10.3390/nursrep12040068

**Published:** 2022-09-27

**Authors:** Lúcia Macedo, Marta Catarino, Constança Festas, Paulo Alves

**Affiliations:** 1 São João University Hospital Center (CHUSJ), EPE, 4200-319 Porto, Portugal; 2 Institute of Health Sciences (ICS), Universidade Católica Portuguesa, 4169-005 Porto, Portugal; 3 Center for Interdisciplinary Research in Health (CIIS), Institute of Health Sciences (ICS), Universidade Católica Portuguesa, 4169-005 Porto, Portugal; 4 Health Department, Polytechnic Institute of Beja, 7800-111 Beja, Portugal; 5 Institute of Health Sciences (ICS), Universidade Católica Portuguesa, 1649-023 Lisboa, Portugal

**Keywords:** children, celiac disease, vulnerability, nursing, scoping review

## Abstract

(1) **Background**: The scientific literature reports that children with celiac disease (CD) are more susceptible to developing physical, psychological and social problems, conditioning their healthy childhood development. Despite this scientific evidence, the knowledge about determinants of vulnerability for the development of such problems is not consistent. In order to search the literature, a scoping review was conducted to analyse and map the evidence on the sociopsychosomatic vulnerability of children with CD and identify the gaps in this topic. (2) **Methods:** The methodology proposed by the Joanna Briggs Institute will be adopted and aims to identify studies that meet pre-defined eligibility criteria. The survey will include a range of relevant electronic databases as well as grey literature using related terms such as vulnerability, child and celiac disease. (3) **Results**: This review will consider any type of quantitative, qualitative and mixed studies and systematic reviews, focusing on dimensions of vulnerability in children with CD. The process of selection of studies, data extraction and analysis will be developed by two independent researchers. A third and fourth researcher will be involved in the study when there is no consensus between the previous researchers, as well as for resolving issues regarding the methodological process. (4) **Conclusions**: Identifying the determinants of vulnerability in children with CD will help nurses to understand the impact on their childhood development and trace possible gaps. This research is registered on the platform Open Science Framework (OSF).

## 1. Introduction

Being a developing child with a chronic health condition is a complex experience which implies constant (re)adaptation. A child with celiac disease (CD) is more likely to develop health problems, since they have a condition that can hinder their healthy childhood development in several aspects (physical, psychological and social), due to the genetic condition predisposition [1,2,3,4,5].

It became evident that CD is a common disease occurring at all ages and with a variety of signs and symptoms. Celiac Disease (CD) is a multifactorial, systemic immune-mediated disorder, in which the HLA immunogenetic background (DQ2 and DQ8 heterodimers—especially HLA-DQB1*02) and environmental trigger (gluten) are well established. Indeed, both factors are necessary, but not sufficient to develop CD [3,4,6].

The ingestion of gluten induces a state of chronic inflammation of the intestinal mucosa that reverts when gluten is excluded, relapsing after its reintroduction in the diet. The only effective treatment is strict adherence to a gluten-free diet (GFD) for life. Adherence to GFD results in the remission of symptoms and intestinal lesions that can re-emerge with non-compliance or involuntary contamination of food [2,3,4,5,7,8].

Currently, the scientific community considers CD to be a public health problem, given its pervasiveness across all nationalities, ethnicities and age groups. CD has been described in the literature as one of the most common chronic diseases in the paediatric population, with an estimated prevalence of 1–2% despite varying prevalence between different countries [8,9,10,11]. Due to increased awareness of CD as well as increased information and evolution of techniques for the morphological, biochemical and genetic study of people with CD [6], more patients will be accurately diagnosed.

Especially in the school setting, where they spend most of their time, children with CD are in a position of greater vulnerability compared to their peers, thus experiencing health inequalities. The disease limits their autonomy [10] as they are dependent on others (either the people responsible for their care at school or their family at home) to meet their special health needs and remove obstacles in the course of their development and healthcare project.

It is noted that some individuals are subject to adverse conditions and develop health problems, while others do not get sick. Each individual must have a vulnerability threshold which results from the interaction between their individual and environmental determinants which, when exceeded, results in the appearance of health problems. Thus, there is an interest in approaching the concept of vulnerability as it precedes the appearance of health problems.

The concept of vulnerability has evolved over the last thirty years. From the analysis of the literature, the relevance of some theoretical frameworks on the definition of the concept of vulnerability is notable [12,13,14,15,16,17], which contributes to a better understanding of this phenomenon [15,18,19,20,21,22].

The authors will use the definition of vulnerability by Rogers: a set of conditions that makes a person more susceptible to adverse health outcomes (physical, psychological, social) as a result of individual and, also, social aspects [12]. The model proposed by Rogers considers that the vulnerability results from the dynamic interaction between their personal resources and the existing environmental support for meeting their health needs [12]. This model is pragmatic, understandable and easy for those who implement it, given the context and characteristics of the target study population.

The literature refers to a considerable number of health consequences of CD in individuals [1,2,5,11]. Although some current reviews focused on the genetic role (and therefore, seeing an evolution of diagnostic techniques and treatment) in the development of disease and associated health problems [3,4,6,7], other factors (individual/environmental) determine a person’s vulnerability, influencing their health status. Consequently, important questions about the nature of the evidence in this area need to be answered before directing nursing interventions in the context of caring for a child with CD.

This mapping will clarify these aspects, hence the decision to conduct a scoping review. By providing a detailed description and summary of the available information, this scoping will contribute to dissemination of research results and also identify possible gaps in knowledge, providing conclusions about the overall state of research activity in this area and need for future research [23].

This scoping review is guided by the Joanna Briggs Institute’s (J.B.I.) methodology for scoping reviews. It aims to map and analyse published scientific evidence on vulnerability in children with CD in different care settings. An initial search of MEDLINE (PubMed), the J.B.I. Evidence Synthesis, the Cochrane Database of Systematic Reviews, PROSPERO and Open Science Framework (O.S.F.) revealed that, currently, there are no scoping reviews or systematic reviews (published or in progress) about this subject [23,24,25].

## 2. Materials and Methods

The final review will be reported according to the guidelines of the Preferred Reporting Items for Systematic Reviews and Meta-Analyses extension for Scoping Reviews (PRISMA-ScR) [26,27].

This has been registered on the Open Science Framework (OSF) platform.

### 2.1. Inclusion Criteria

Based on the JBI recommendations in the PPC mnemonic guide *What evidence has been published regarding the vulnerability of children with CD in different health care settings?* for the scoping review, the inclusion criteria are related to:

- participants—studies looking at children with CD (pre-school, school and adolescent age);
- concept—this review will consider studies that explore vulnerability;
- context—this review will consider studies, regardless of the country of study, conducted in any clinical practice setting;

Furthermore, this review intends to answer the following sub-questions:

- What is the published evidence on the dimensions/issues/characteristics of vulnerability in children with CD?
- What other concepts are related to the concept of vulnerability in children with CD?
- Is there evidence on tools to assess vulnerability?
- Is there published evidence on the situations, circumstances and conditions/factors/determinants that positively or negatively influence vulnerability in children with CD?

The body of the literature will be composed of any study designs with quantitative, qualitative and mixed methodology editorial letters. Mixed studies as well as grey literature (theses and dissertations) are also expected to be included. Furthermore, all types of systematic reviews will be considered in the proposed review.

Only documents in Portuguese, Spanish or English will be considered, without limitation as to the period of publication.

Other published manuscripts that the authors consider relevant to include in this analysis may also be included, provided that they meet the eligibility criteria.

### 2.2. Search Strategy

The search strategy will find published and unpublished primary studies and reviews. Two reviewers will develop the search strategy, which will be peer reviewed by the third expert reviewer the basis of the Peer Review of Electronic Search Strategies (PRESS) [28].

The three-step search strategy recommended by J.B.I. was applied [23,24].

A limited preliminary search was initiated in MEDLINE (via PubMed) with the aim of investigating the words in the text included in the title and abstract, as well as the index terms used to describe the selected studies. Subsequently, a full search strategy was developed (Table 1). The search was carried out on 28 March 2022.

It is emphasized that the search strategy will be adapted to the specificities of each information source. Finally, the reference lists of the articles included in the review will be selected for supplementary articles.

The languages of the articles will be restricted to those understood by the authors—English, Spanish and Portuguese—to ensure a good quality selection and data extraction procedure. Articles written in other languages will be excluded on the basis of language. However, they will be reported on for transparency in the scoping review report.

The full search will include the electronic databases Cumulative Index to Nursing and Allied Health Literature (CINAHL) (via EBSCOhost), Medical Literature Analysis and Retrieval System online (MEDLINE) (via PubMed), Cochrane Library, Scielo, Literatura Latino-Americana e do Caribe em Ciências da Saúde (LILACS), Scopus and Web of Science, as these are databases with peer-reviewed publications (of a qualitative and quantitative nature), thus enabling the attainment of the proposed objective. An electronic search of dissertation and thesis abstracts will also be conducted in the Scientific Open Access Scientific Repositories of Portugal (RCAAP) and ProQuest Dissertations and Theses, Biblioteca Digital Brasileira de Teses e Dissertações (BDTD) databases, since they relate to unpublished studies.

### 2.3. Study Selection

Note that all identified citations will be exported to EndNote Web software (Clarivate Analytics, PA, USA). Duplicates are removed in this process.

Article titles and abstracts will be screened for eligible criteria by two independent researchers using the Rayyan QCR platform.

A pilot test will be conducted to verify that the inclusion criteria are being met. At this stage, full-text articles will be read and examined according to the defined criteria.

From the identification of relevant articles, data will be extracted using a standardised form and assessed.

Subsequently, the list of references of all studies selected for critical appraisal will be further analysed. This step aims to check for the existence of additional studies not previously identified. It may be necessary to contact specialists in the area to collaborate in the search, in order to contribute their expertise. It may also be necessary to contact the authors of the studies identified for possible clarifications or to provide references.

The reasons for exclusion of studies that do not meet the eligibility criteria will be recorded and reported.

Each phase of the research will have the participation of two independent reviewers to systematize this review and reduce research bias, taking into account the pre-established inclusion criteria and the research question. In the case of a disagreement between them, a third reviewer will be consulted in order to at least establish agreement between two reviewers regarding the selected articles.

### 2.4. Data Extraction

The data extraction of the articles included will be undertaken by two independent reviewers, using a form that considers specific details about the population, the concept, the context and the research methods relevant to the question and the stated objective of this scoping review, as indicated by the methodology developed by JBI (Table 2).

It is worth noting that the authors will conduct a pilot test of this form before starting extraction, and adjustments may be made to the data extraction tool during the review process [29].

### 2.5. Data Analysis and Presentation

Data will be presented graphically or in schematic or tabular form. A narrative summary of the findings of the studies included will be prepared. This summary will provide information on the mapping of the studies carried out around this theme, taking into account the purpose of this review [23,24,26] and a qualitative evaluation of the data analysis.

## 3. Discussion

Considering that the concept of vulnerability has evolved and is expanding, it is considered important to conduct a brief historical review of its evolution.

In the 1980s, vulnerability referred to the term applied within the epidemiological concept of risk that consisted of the probability that an individual had to become ill within a period of time [13]. These researchers began to analyse the concept of vulnerability, also investigating its main determinants. From the authors’ perspective, risk and vulnerability are the individual factors (innate and/or acquired) that, when interacting with each other, determine their vulnerability and influence the health results obtained.

Less well known is that the vulnerability theory proposed by Lessick and collaborators admits that the level of vulnerability of individuals is dynamic and can be altered according to changes in the person, in the environment (or in both), and that each individual has a threshold of vulnerability that, when overcome, results in the appearance of the disease [14].

In the 1990s, the concept of vulnerability was expanded, with the contributions of Aday. He emphasized the health care needs of nine population groups, identifying those who are at increased risk for poorer physical, psychological and/or social health [18]. His work consisted of the elaboration of a conceptual, empirical and normative reference point to understand the origins and consequences of poor health, allowing it to guide the development of research and political priorities with a view to meeting the health needs of the growing number of vulnerable populations [19].

Progressing in the chronological evolution, in 1997, Ayres considered that risk and vulnerability have a close relationship and are important in the interpretation of the health–disease process: risk indicates probabilities, and vulnerability precedes risk and determines the different risks of falling ill for an individual, thus expressing its potential for (non) illness as a carrier of specific characteristics [20,21].

In the same year, Rogers proposes that the degree of vulnerability results from the interaction of personal and environmental resources, based on the contributions of Aday, Rose and Killien, Aday and Lessick and collaborators for defining the personal and environmental determinants of vulnerability [12].

In the literature, the Conceptual Model of Nursing for Vulnerable Populations that stood out the most was the one developed by Flaskerud and Winslow, defining vulnerability as the complex interaction between risk, susceptibility, availability of resources and health status [22].

In 2003, Dorsey and Murgaugh proposed a middle-range theory, entitled Self-Care Management Theory for Vulnerable Populations, which suggests an alternative approach to the concept of vulnerability, focusing on the management of self-care and intrapersonal factors to manage diseases, with this management being dependent on contextual factors. Individuals with chronic disease find modifiable and non-modifiable factors that can increase their vulnerability [15].

As the concept is clarified, the more it becomes fluid in the literature, witnessing a shift to a more open, less stigmatizing, potentially expansive meaning [16] and acquiring a sense of “opportunity for”. Thus, resources, resilience and adaptability stand out as key factors in the results associated with vulnerability [17].

This overview was essential in determining key search terms, given that the concept of vulnerability has been studied for the past 30 years, so that many articles addressing aspects of vulnerability were included in this review. Publications will be considered, with no limitation in time, in the search strategy.

The scoping review will only consider studies in English, Portuguese and Spanish, which may be registered as a potential limitation of the study. To address this limitation, abstracts of articles published in other languages considered relevant may be included, through translation in Google Translator and Linguee, to avoid restrictions to specific programs for certain cultures.

## 4. Conclusions

It is agreed that the concept of vulnerability is expanding, which makes its assessment difficult. In this sense, this review will help researchers to identify which factors/characteristics/dimensions of vulnerability have been studied in the academic literature. All information will be important for the development of the knowledge and growth of the nursing discipline. This analysis and systematization will allow the identification of gaps for future investigations.

In conclusion, this scoping review will examine emerging evidence and provide an overview of this important topic. It makes sense to consider that, at the end of this review, relevant data will emerge (individual and environmental determinants/factors) that can contribute to the development of a tool to stratify the vulnerability risk, as well as their potential for coping and for directing nursing interventions.

## Figures and Tables

**Table 1:** Search strategy used in one of the databases—MEDLINE (via PubMed).

Search	Query ^1^	Records Retrieved
#1	(vulnerab*[MeSH Terms]) OR (“vulnerab*”[Title/Abstract])	4409
#2	(“vulnerab*”[Title/Abstract])	107,826
#3	“vulnerab*”[Title/Abstract] OR “risk”[Title/Abstract] OR “susceptib*”[Title/Abstract]	3,054,384
#4	(“celiac disease”[Title/Abstract] OR “coeliac disease”[Title/Abstract] OR “celiac sprue”[Title/Abstract] OR “gluten-sensitive enteropathy”[Title/Abstract])	20,641
#5	“celiac disease” [MeSH Terms]	21,480
#6	(“child*” OR “adolescen*”[MeSH Terms])	4,188,053
#7	(Child*[Title/Abstract] OR adolescen*[Title/Abstract] OR infan*[Title/Abstract] OR teen*[Title/Abstract] OR youth[Title/Abstract] OR scholar[Title/Abstract] OR pediatric[Title/Abstract] OR paediatric [Title/Abstract])	2,336,753
#8	#3 AND #4 AND #7	1207

^1^ Limited to language (English, Portuguese, Spanish).

**Table 2:** Data extraction tool.

**Details of the Scoping Review**	
**Title of the Scoping Review**: Vulnerability in children with celiac disease: a scoping review protocol	
**Goals**: to analyse the literature and map the scientific evidence regarding the vulnerability of children with celiac disease in different health care settings.	
**Research Question**: “What evidence has been published regarding the vulnerability of children with CD in different health care settings?” Sub-questions: - What is the published evidence on the dimensions/issues/characteristics of vulnerability in children with CD? - What other concepts are related to the concept of vulnerability in children with CD? - Is there evidence on tools to assess vulnerability? - Is there published evidence on the situations, circumstances and conditions/factors/determinants that positively or negatively influence vulnerability in children with CD?	
**Eligibility Criteria**	
- Participants: The review will consider studies that include school-aged (between the ages of 6 and 19) children and adolescents with CD. - Concept: Studies that will explore vulnerability. - Context: Studies of a multidisciplinary nature, in different areas of expertise (hospital, primary health care, among others) will be included. No cultural or geographical restrictions.	
**Characteristics of Source of Evidence**	
Article Code/Database	
Citation details (author/s, date, title, magazine, volume, editing, pages), Country, Language	
Scientific discipline	
Study objectives	
Context	
Participants (population and sample size)	
Study design/Methodology/Level of evidence	
**Results Extracted from the Source of Evidence**	
Study results (Vulnerability aspects studied/ Concepts related to vulnerability/Determinants/conditions/circumstances/situations that influence vulnerability)	
Limitations	
Future Research Recommendations/Perspectives	
Bibliography cited	
Comments	

## Data Availability

Not applicable.
